# Viscoelastic and ultrastructural characteristics of whole blood and plasma in Alzheimer-type dementia, and the possible role of bacterial lipopolysaccharides (LPS)

**DOI:** 10.18632/oncotarget.6074

**Published:** 2015-10-10

**Authors:** Janette Bester, Prashilla Soma, Douglas B. Kell, Etheresia Pretorius

**Affiliations:** ^1^ Department of Physiology, Faculty of Health Sciences, University of Pretoria, Arcadia, South Africa; ^2^ School of Chemistry and The Manchester Institute of Biotechnology, The University of Manchester, Manchester, UK

**Keywords:** Alzheimer-type dementia, iron levels, lipopolysaccharides, scanning electron microscopy, thromboelastography®, Gerotarget

## Abstract

Alzheimer-type dementia (AD) is a neurodegenerative disorder and the most common form of dementia. Patients typically present with neuro- and systemic inflammation and iron dysregulation, associated with oxidative damage that reflects in hypercoagulability. Hypercoagulability is closely associated with increased fibrin(ogen) and in AD patients fibrin(ogen) has been implicated in the development of neuroinflammation and memory deficits. There is still no clear reason precisely why (a) this hypercoagulable state, (b) iron dysregulation and (c) increased fibrin(ogen) could together lead to the loss of neuronal structure and cognitive function. Here we suggest an alternative hypothesis based on previous ultrastructural evidence of the presence of a (dormant) blood microbiome in AD. Furthermore, we argue that bacterial cell wall components, such as the endotoxin lipopolysaccharide (LPS) of Gram-negative strains, might be the cause of the continuing and low-grade inflammation, characteristic of AD. Here, we follow an integrated approach, by studying the viscoelastic and ultrastructural properties of AD plasma and whole blood by using scanning electron microscopy, Thromboelastography (TEG^®^) and the Global Thrombosis Test (GTT^®^). Ultrastructural analysis confirmed the presence and close proximity of microbes to erythrocytes. TEG^®^ analysis showed a hypercoagulable state in AD. TEG^®^ results where LPS was added to naive blood showed the same trends as were found with the AD patients, while the GTT^®^ results (where only platelet activity is measured), were not affected by the added LPS, suggesting that LPS does not directly impact platelet function. Our findings reinforce the importance of further investigating the role of LPS in AD.

## INTRODUCTION

Alzheimer-type dementia (AD) is a neurodegenerative disorder and the most common form of dementia [1–3]. Dementia is a syndrome applied to a group of symptoms that can be caused by a variety of conditions, the most common of which is Alzheimer's disease; unfortunately, due in part to this ambiguity, the aetiology of AD is not well understood [4]. The onset and risk of -development is still mostly unexplained, and only very partially so by genetic factors [4]. Today AD is the largest unmet medical need in neurology [5–7], and is a condition characterized by neuroinflammation [8, 9]. Neuroinflammation is a multi-faceted and complex phenomenon where the precise mechanisms have not been completely elucidated [10], but where activated astrocytes and microglia are usually the trigger in the neuroinflammatory process. They become reactive in response to virtually all pathological situations in the brain such as axotomy (neuritic dystrophy), ischemia, infection, and existing neurodegenerative diseases [11–15].

**Figure 1:**
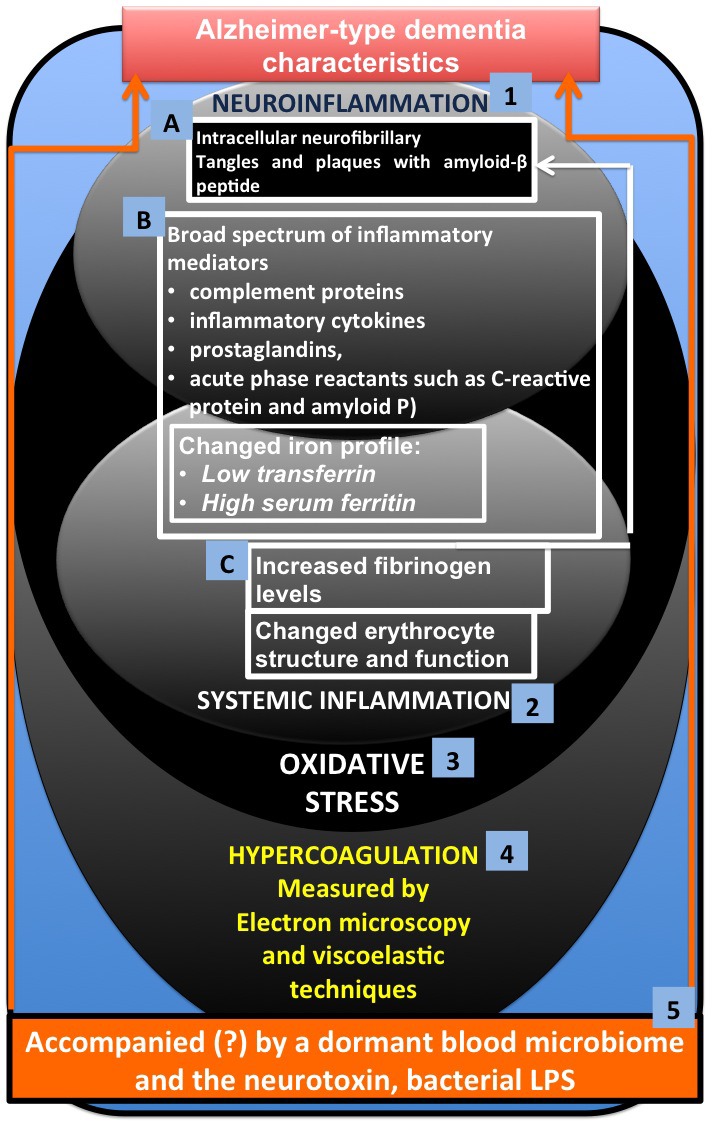
Alzheimer-type dementia (AD) and three of its main characteristics (1) neuroinflammation (2) systemic inflammation and (3) oxidative stress, where A involves specific changes in brain morphology and **B.** shows shared inflammatory mediators between neuroinflammation and systemic inflammation (with special reference to a changed iron profile) and **C.** factors that are typically implicated in systemic inflammation, but that have an impact on the brain. The three main characteristics are affected by and also result in (4) hypercoagulation, which we can measure using electron microscopy and viscoelastic techniques. We conclude by discussing how the presence of a dormant blood microbiome, and in particular the lipopolysaccharides (LPS) and other cell wall materials that they can shed, may be central to the hypercoagulable/neuroinflammatory state in this condition.

In this paper, we discuss the factors involved in systemic inflammation and show that they are also typically prevalent in neuroinflammation. We focus specifically on literature that shows a changed iron profile, increased fibrinogen levels and oxidative stress; these have each been implicated in a typical hypercoagulable (thrombotic) state, but are also present in AD (the literature is discussed fully in the different sections). Figure 1 shows the layout of this paper, where the focus is on 3 main characteristics of AD, namely (1) neuroinflammation (2) systemic inflammation and (3) oxidative stress. We briefly discuss specific changes in brain morphology (A) and shared inflammatory mediators between neuroinflammation and systemic inflammation (B) (with special reference to a changed iron profile). Factors that are typically implicated in systemic inflammation, but that have an impact on the brain (C) are then examined. We then consider how the three main characteristics are affected by and also result in (4) hypercoagulation and how we can measure this by using electron microscopic and viscoelastic techniques. Lastly, we conclude by discussing possible reasons for this hypercoagulability in AD, and we discuss that the presence of a dormant blood microbiome, and in particular the lipopolysaccharides (LPS) that they can shed, may be central to the hypercoagulable state in this condition [16, 17].

## NEUROINFLAMMATION AND THE IRON LINK

The next paragraphs will briefly discuss presentation of neuroinflammation and the involvement of iron and its accompanying oxidative damage in AD etiology. We will make the link between the presence of hypercoagulability in AD and oxidative damage, changed iron levels and inflammation (both neuro- and systemic inflammation).

The hallmarks of dementia in AD, as well as the severity and state of neurodegeneration, are closely linked to the presence of tau phosphorylation, amyloid-β peptide aggregation, neurofibrillary tangle formation, neuroinflammation, and neurodegeneration [18, 19]. If we look closely at the presentation of AD, it is characterized by brain lesions known as intracellular neurofibrillary tangles and extracellular neuritic plaques surrounded by activated astrocytes and microglia [20]. The neurofibrillary tangles consist of paired helical filaments of truncated tau protein that is abnormally hyperphosphorylated [21, 22]. The main component in the plaques is the amyloid-β peptide [1, 23], though its role in the actual disease pathology is more than questionable [24]. The brain lesions in AD are also characterized by the presence of a broad spectrum of inflammatory mediators (complement proteins, inflammatory cytokines, prostaglandins, and acute phase reactants such as C-reactive protein and amyloid P) [25, 26]. Resident brain cells, including neurons produce these mediators [27, 28]. Neuroinflammation is therefore key in AD [26, 28–33]. Central to this neuroinflammation in AD is the involvement of iron and its accompanying oxidative damage in AD etiology [34–48]. Oxidative damage is one of the earliest pathological changes in AD, and the aberrant redox activity is therefore also among the earliest changes in the transition to the disease state [49]. Note that as long ago as 1991 it was shown that chelators of free iron improved cognitive function in AD sufferers [40, 50].

## OXIDATIVE DAMAGE, IRON, INFLAMMATION AND HYPERCOAGULABILITY

Oxidative damage, increased iron levels and inflammation are all linked to the development of hypercoagulability [51–58]. Increased fibrin(ogen) levels have also been noted in hypercoagulation [53, 59–65] and this is also observed in blood vessels positive for amyloid in mouse and human AD samples [66]. Fibrinogen extravasation in the AD brain has also been noted [67]. Thus, fibrin(ogen) may play an important role in AD etiology as it has been implicated in the neuroinflammation, neurovascular damage, blood-brain barrier permeability, vascular amyloid deposition, and memory deficits, all of which are associated with AD [68]. Increased fibrinogen levels in AD are therefore a strong cerebrovascular risk factor in these patients, as fibrinogen specifically binds to β-amyloid, thereby altering fibrin clot structure and delaying clot degradation [69]. However, there is currently no evidence regarding the extent to which delayed clot degeneration might contribute to AD.

**Figure 2:**
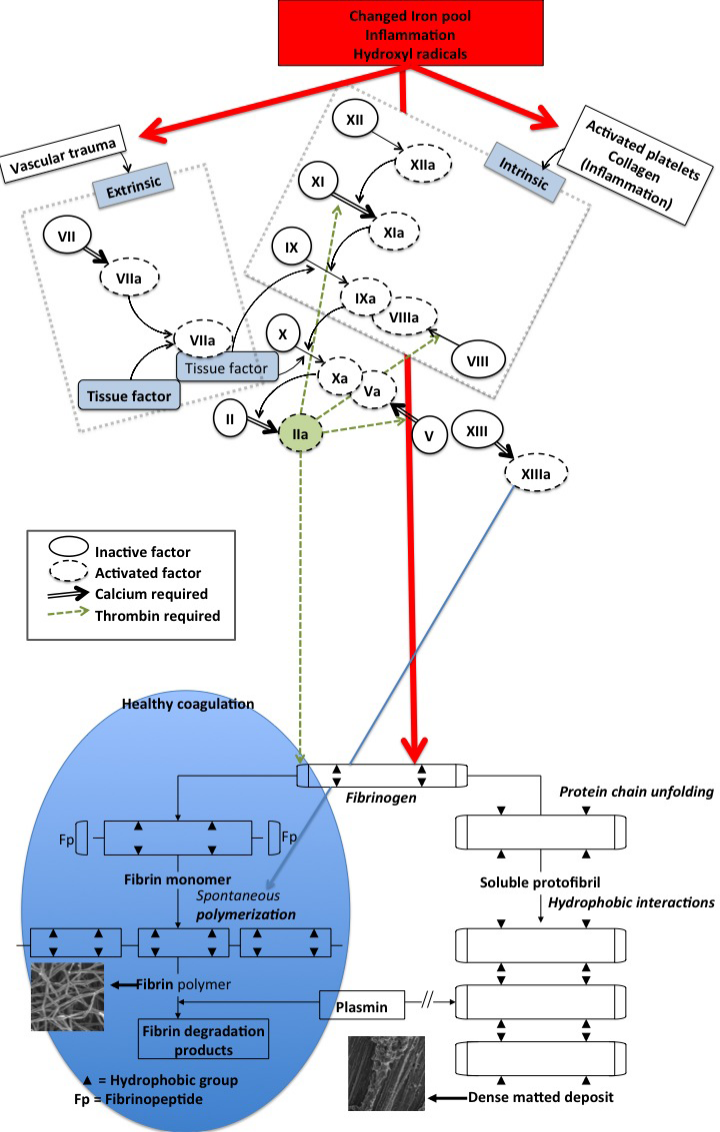
Schematic representation of the intrinsic and extrinsic coagulation pathway and the conversion of soluble fibrinogen into insoluble polymers Thrombin‘s action on fibrinogen results in the formation of fibrin strands (left insert) that are in time degradable with plasmin. By contrast, iron-induced dense matted deposits (right insert) are less degradable [89] - this is during normal blood coagulation. Iron-induced blood coagulation is seen as dense matted deposits under scanning electron microscopy and viscoelastically [85, 87, 88].

Thrombosis, and therefore hypercoagulation, is typically associated with increased levels of fibrin(ogen) [58, 70, 71]. A changed iron profile (e.g. low transferrin and/or high serum ferritin) is also central to thrombosis and hypercoagulation [38, 58, 72–79] and iron dysregulation is heavily implicated in AD [38, 80]. Changes in fibrin fibre structure (due to hypercoagulation and a changed iron profile) can be visualized using various ultramicroscopy techniques [54, 58, 81–86]. In addition, these changes might be correlated with results from viscoelastic techniques like thromboelastography (TEG^®^) [52, 79, 85, 87, 88]. Figure 2 shows how the intrinsic and extrinsic coagulation process causes fibrin fibre formation under normal conditions, and how fibrin fibre formation is changed under the influence of a changed (aberrant) iron profile, whether by generating hydroxyl radicals (resulting in oxidative stress, and also during systemic inflammation) [89] or by electrostatic means [57, 58].

## LINK BETWEEN A HYPERCOAGULABLE STATE, IRON DYSREGULATION, CHANGES IN FIBRIN FIBRE STRUCTURE AND A STERILE MICROBIOME

This said, there is still no clear reason precisely why (a) this hypercoagulable state, (b) iron dysregulation and (c) changes in fibrin fibre structure could together lead to the loss of neuronal structure and cognitive function in AD. The following paragraphs will discuss the link between (a to c) and a supposedly sterile blood microbiome.

Recently, we showed (with ultrastructural microscopy techniques) that, in the “sterile” blood microbiome of AD patients, there are bacteria present, that we have suggested are normally dormant [16, 90]. Dormancy is in fact the norm in environmental and general microbiology [91, 92]. We note that there is a substantial literature implicating chronic infection [93–97] as well as the gastrointestinal tract microbiome in AD [98–100]. In particular, periodontal disease related pathogens and their inflammatory products have been shown to contribute to, or at least to accompany, systemic inflammation and the pathogenesis of AD [93, 101–105]. Particularly, periodontal pathogens *Porphyromonas gingivalis*, *Tannerella forsythia*, and *Treponema denticola* have been implicated in the development of AD [106, 107]. Infections with *Herpes simplex* virus type 1 [97], picornavirus, Borna disease virus, *Chlamydia pneumoniae*, *Helicobacter pylori*, and spirochetes [96, 108] and pathogens causing urinary tract infections [94, 109–111] are also implicated and co-occurring in AD.

Recently, immunoblotting demonstrated bands corresponding to lipopolysaccharides (LPS) (also known as endotoxin), produced or shed by *P. gingivalis* in 40% of AD brain specimens [107]. Indeed, there is evidence that bacterial endotoxins are directly involved in the inflammatory and pathological processes associated with AD [112]. Interestingly, it has been observed that chronic infusion of the bacterial LPS, the outer cell wall component of Gram negative bacteria, into the fourth brain ventricle of rats reproduces many of the inflammatory and pathological features seen in the brain of AD patients [112]. Apolipoprotein E (ApoE) is an intermediate-density lipoprotein that is essential for the normal catabolism of triglyceride-rich lipoprotein constituents. It also transports cholesterol (its primary function), and regulates amyloid-β (Aβ) metabolism, aggregation, and deposition [113]. Interestingly, LPS may disturb the typically anti-inflammatory effect of ApoE. ApoE function is known to be down-regulated in AD patients [114, 115], and carrying the ApoE4 allele increases risk of Alzheimer's disease [113, 116, 117]. The down-regulating effect on ApoE by LPS was also seen in an animal model [118]. Furthermore, LPS is used in animal models to induce AD-like symptoms, as well as neuroinflammation [119, 120], as well as Parkinson's disease-like symptoms [17, 121–128]. Recently it was also noted that LPS can produce myelin injury and plaque-like aggregates of myelin in mice, and amyloid-β and amyloid-β protein precursor co-localize with these myelin aggregates. Cortical amyloid plaques also co-localized with myelin aggregates [129]. Following LPS injection, Alzheimer-like amyloidogenic axonal pathology also occur in the normal mammalian brain in partnership with neuroinflammation [130], and LPS is associated with the development of neuroinflammation in animals [130], as well as in primary culture neuroinflammatory models [131].

From the above, and other literature reviewed elsewhere [17, 132], there is ample evidence that LPS can cause neuroinflammation and amyloid-β formation. There is also evidence for the involvement of LPS in cognitive impairment in AD. For instance,

- LPS is used to induce cognitive impairment in mice [120, 133].
- LPS induces stress and depression in late onset AD [134].
- Neuro-inflammation, amyloidogenesis and memory impairment in a mouse model is seen following the systemic inflammation generated by LPS [120].
- Intraperitoneal injection of LPS causes attention deficits [135] and severe and fluctuating cognitive deficits in 16-week ME7 mice [136].

Where does this bacteria/LPS argument lead us to? It is known that LPS can also cause hypercoagulation [137, 138]; this has been referred to as endotoxin-mediated hypercoagulation [139]. There is substantial evidence in the literature that microbes have a prominent involvement in AD, but it seems that only a few researchers have taken the final step to suggest that such bacteria may actually cause or exacerbate AD (in the sense of “Koch's postulates”) [96], and that shedding of LPS from the (dormant) blood microbiome may exacerbate or even cause the hypercoagulability seen in AD. Clearly the measurement of hypercoagulability is considerably easier than the estimation of cognitive function.

In the current paper we used scanning electron microscopy, together with viscoelastic techniques, to study whole blood and plasma in AD patients and compared the results with those of age- and gender-matched healthy individuals. We also studied iron profiles and general hematological parameters. Lastly, we simulated the effect of “physiological” levels of LPS, by adding it to healthy, uncitrated blood. We determine if LPS has an effect on platelets by using a novel technique called the Global Thrombosis Test (GTT) [140, 141]. This allows for the detection of thrombin generation by activated platelets, the major determinant of arterial thrombogenesis and measured endogenous (spontaneous) thrombolytic activity (http://www.globalthrombosis.com). Typical LPS concentrations in ‘normal’ whole blood in healthy subjects seem to be of the order of 10-15 ng.L^−1^ [17, 142, 143], while those of LBP are roughly 1,000,000 times greater at 5-15 mg.L^−1^ (with both values increasing during sepsis). The exact significance of these numbers is not clear [17] as LPS is so hydrophobic that most is bound to the LPS-binding protein or lipoproteins [144–146]. We also added LPS to naïve whole blood and measured coagulation parameters with the TEG^®^, to determine directly if added LPS causes hypercoagulability. If this were to be the case, it would imply that LPS can bind directly to fibrinogen.

## RESULTS

### Healthy individual and Alzheimer-type dementia patient data

Table 2 show demographics for healthy and AD individuals, iron profiles, and data for the TEG^®^, GTT^®^ and SEM.

**Table 1:** Thromboelastograph® parameters typically generated for whole blood and platelet poor plasma [153, 154]

THROMBOELASTIC PARAMETERS		
R value: reaction time	Minutes	Time of latency from start of test to initial fibrin formation (amplitude of 2mm); i.e. initiation time
K: kinetics	Minutes	Time taken to achieve a certain level of clot strength (amplitude of 20mm); i.e. amplification
A (Alpha): Angle (slope between the traces represented by R and K)	Angle in degrees	The angle measures the speed at which fibrin build up and cross linking takes place, hence assesses the rate of clot formation; i.e. thrombin burst
MA: Maximal Amplitude	mm	Maximum strength/stiffness of clot. Represents the ultimate strength of the fibrin clot, i.e. overall stability of the clot
Maximum rate of thrombus generation (MRTG)	Dyn.cm^−2^.s^−1^	The maximum velocity of clot growth observed
Time to maximum rate of thrombus generation (TMRTG)	Minutes	The time interval observed before the maximum speed of the clot growth
Total thrombus generation (TTG)	Dyn.cm^−2^	The clot strength: the amount of total resistance (to movement of the cup and pin) generated during clot formation. This is the total area under the velocity curve during clot growth representing the amount of clot strength generated during clot growth

**Table 2:** Demographics of participants, as well as iron levels, thromboelastography® (TEG®) of plasma, fibrin fiber thickness, and thromboelastography® (TEG®) and global thrombosis test (GTT®) of naïve blood of age-matched controls (without dementia) and Alzheimer-type dementia (AD) patients, showing medians, standard deviation and p-values (values lower than 0.05 are indicated in blue) obtained using the Mann-Whitney U test

Variables	Healthy individuals (n = 20)	Alzheimer-type dementia individuals (n = 40)	*P*-value	Confidence interval (95%) relative to median
AGE years	68.5 (±22.21)	79 (±11.8)	0.316	−4 to 21
GENDER				
Male	6 (40%)	12 (30%)		
Female	14 (60%)	28 (70%)		
IRON PROFILES				
Iron μM	18.8 (± 5.74)	13.55 (±4.98)	0.0293	0.4 to 6.8
Transferrin g.L^−1^	2.55 (± 0.39)	2.11 (± 0.39)	0.0001	0.2 to 0.7
% Saturation	28 (± 10.32)	26 (± 9.95)	0.6543	−5 to 8
Serum Ferritin ng.mL^−1^	79 (±78.39)	99 (±128.84)	0.457	−59 to 21
**THROMBOELASTOGRAPHY® OF PLATELET POOR PLASMA**				
MRTG	5.35 (± 2.83)	8.47 (± 4.82)	0.0013	1.21 to 4.79
TMRTG	9.46 (± 4.24)	7.79 (± 5.02)	0.0428	0.08 to 2.67
TTG	243.89 (± 85.66)	283.97 (± 116.51)	0.157	−97.47 to 16.3
R	7.2 (± 3.60)	6.2 (± 4.18)	0.122	−0.2 to 2
K	2.35 (± 0.73)	1.4 (± 4.23)	0.0015	0.3 to 1.2
Angle	66.85 (± 9.60)	74.05 (± 8.64)	< 0.0001	−10.6 to −3.9
MA	32.7 (± 8.96)	36.1 (± 9.67)	0.15	−8.3 to 1.4
FIBRIN FIBRE THICKNESS	n = 1000	n = 2000		
Fibre thickness in nm	110 (± 33.8)	115 (± 44.18)	<0.0001	6 to 12
**TEG® RESULTS OF NAÏVE WHOLE BLOOD WITH AND WITHOUT ADDED LPS**				
	Healthy Whole Blood(n = 10)	Healthy Whole Blood with added LPS(n = 10)		
MRTG	2.42 (± 0.41)	2.835 (± 0.67)	0.404	−0.82 to 0.44
TMRTG	15.04 (± 2.98)	11.835 (± 1.92)	0.0003	1.66 to 6.25
TTG	605.21 (± 122.53)	620.89 (± 133.94)	0.739	−135.27 to 81.88
R	10.2 (± 1.01)	7.1 (± 1.82)	0.001	1.8 to 3.9
K	5.35 (± 1.08)	5 (± 1.28)	0.252	−0.6 to 1.7
ANGLE	44.8 (± 4.86)	49.1 (± 2.08)	0.085	−6.5 to 1.1
MA	53.65 (± 5.80)	55 (± 5.13)	0.616	−4.9 to 3.1
**GTT® RESULTS OF NAÏVE WHOLE BLOOD WITH AND WITHOUT ADDED LPS**				
	Healthy Whole Blood(n = 10)	Healthy Whole Blood with added LPS(n = 10)		
OT	335.8 (± 80.96)	354.35 (± 73.66)	0.481	−101.8 to 49
LT	1576 (± 322.24)	1534.5 (± 155.19)	0.315	−93 to 369

Literature suggests that normal values for serum iron are between 11.6 and 31.4 μmol.L^−1^ [158], for transferrin they are 2.2 - 3.7 g.L^−1^ [158], normal % saturation is 20-50% [158], and serum ferritin for males are between 25 - 300 μg.L^−1^ and females are between 25 - 200 μg.L^−1^ [159]. However, values for normal ranges vary between pathology laboratories. Our values are therefore taken according to the values for South Africa (http://ampath.co.za) and indicated in the table. In the current sample, the iron and transferrin showed significant differences between the healthy and AD population, with the transferrin for the AD individuals being mostly lower (see Table 2).

In our previous paper on the hematological system and systemic inflammation of AD, we reviewed the importance of increased iron, and particularly serum ferritin [160]. Results from the current AD sample show that both iron and transferrin levels were significantly different between the healthy and AD individuals. Transferrin is lowered in inflammation [72, 161], and is known as a “negative” acute-phase protein [161]; it is also decreased in in AD [162]. In the present sample most of the AD individuals have serum ferritin levels within the normal ranges for their genders.

### Thromboelastography^®^ results

The following parameters were all changed in the AD patients, and showed a significant difference between the healthy and AD individuals:

- Increased maximum rate of thrombus generation (MRTG),
- Decreased time to maximum rate of thrombus generation (TMRTG) which is the time interval (s) observed before maximum velocity of clot growth,
- Shorter time taken to achieve final clot strength i.e. amplification (K)
- as well as an increased Alpha or Angle representing the speed at which the fibrin build-up and crosslinking of the fibrinogen fibres takes place, or therefore to stabilize the clot.

The results are in line with a faster initial clot formation but and increased time to stabilize the clot in AD compared to controls.

### Scanning electron microscopy (SEM) of whole blood and platelet poor plasma (PPP) results

**Figure 3:**
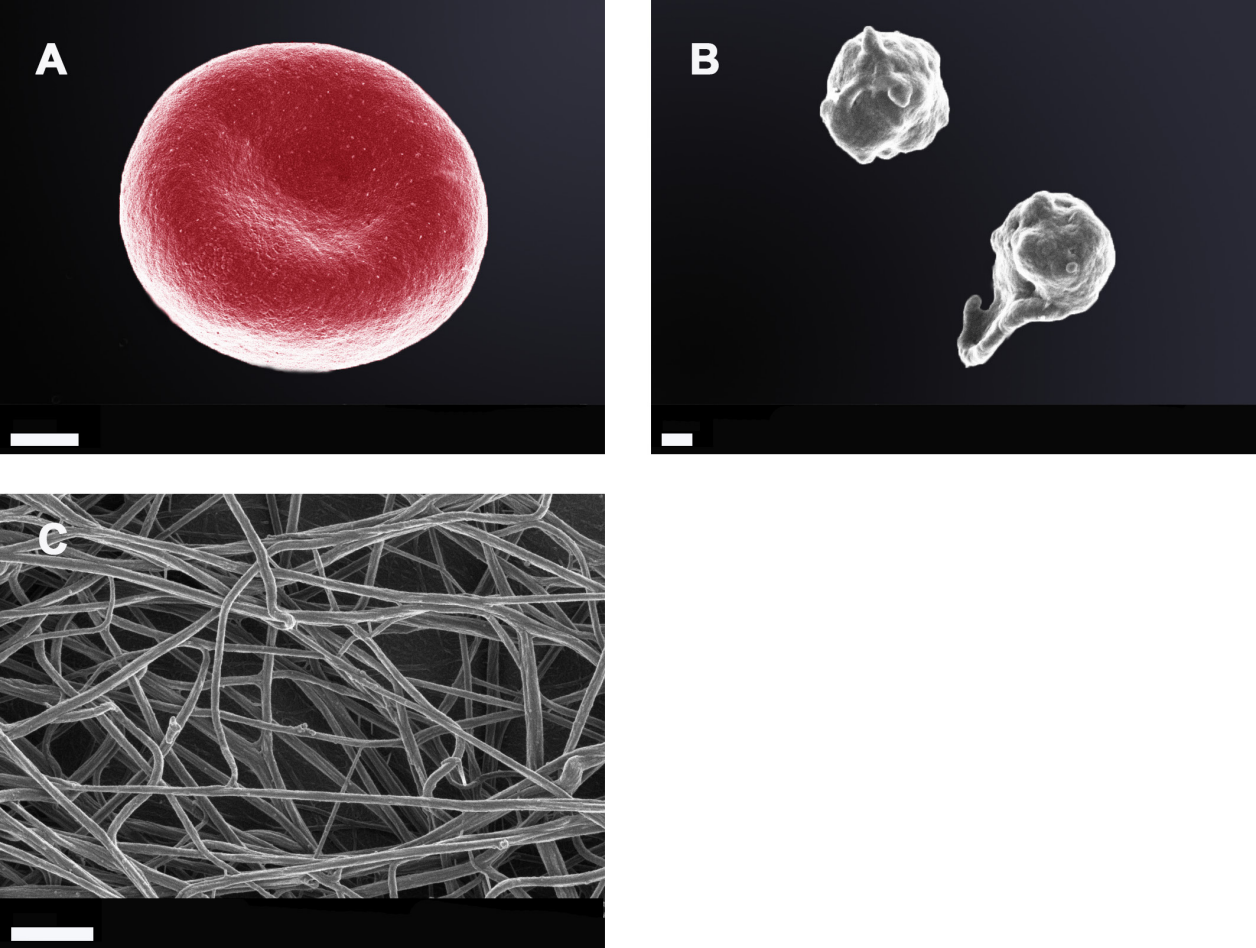
**Whole blood smear showing a typical erythrocyte; Scale bar: 1 μm A.;** two healthy platelets with slight pseudopodia formation; Scale bar: 300 nm **B.**; and an extensive fibrin network created by adding thrombin (58 nM thrombin final concentration with plasma) to platelet poor plasma; Scale bar: 1 μm **C.**

**Figure 4:**
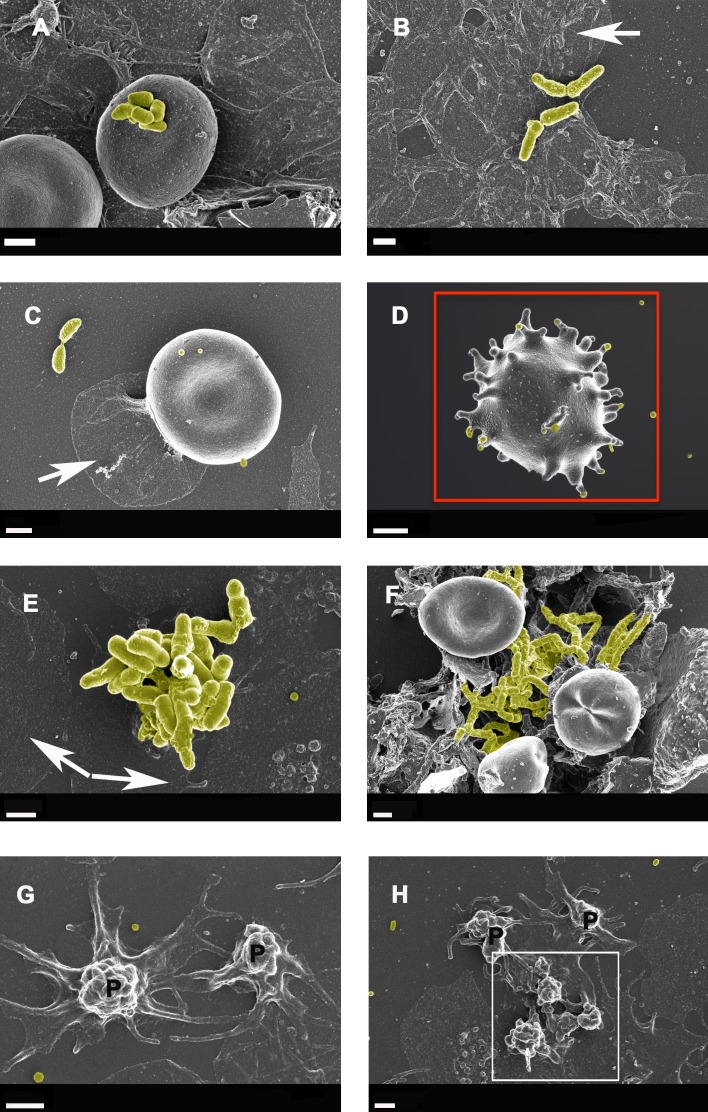
Whole blood smear showing erythrocyte and platelet interactions from Alzheimer-type dementia individuals, with bacterial presence **A.** to **F.** Hyperactivated platelets with spreading are shown in **G.** and **H.** White arrows show platelet spreading, indicative of inflammation and hyperactivation - this is known to happen in all inflammatory conditions; for a discussion on hyperactivation of platelets see [166]. Blue arrows show matted plasma/fibrin deposits. False yellow colouring was added to emphasize the presence of microbiota. Red block shows eryptotic erythrocyte. P shows partially intact but activated platelets, where spreading is starting happen, and white block surrounds such an activated platelet mass. Scale bars: 1 μm.

**Figure 5:**
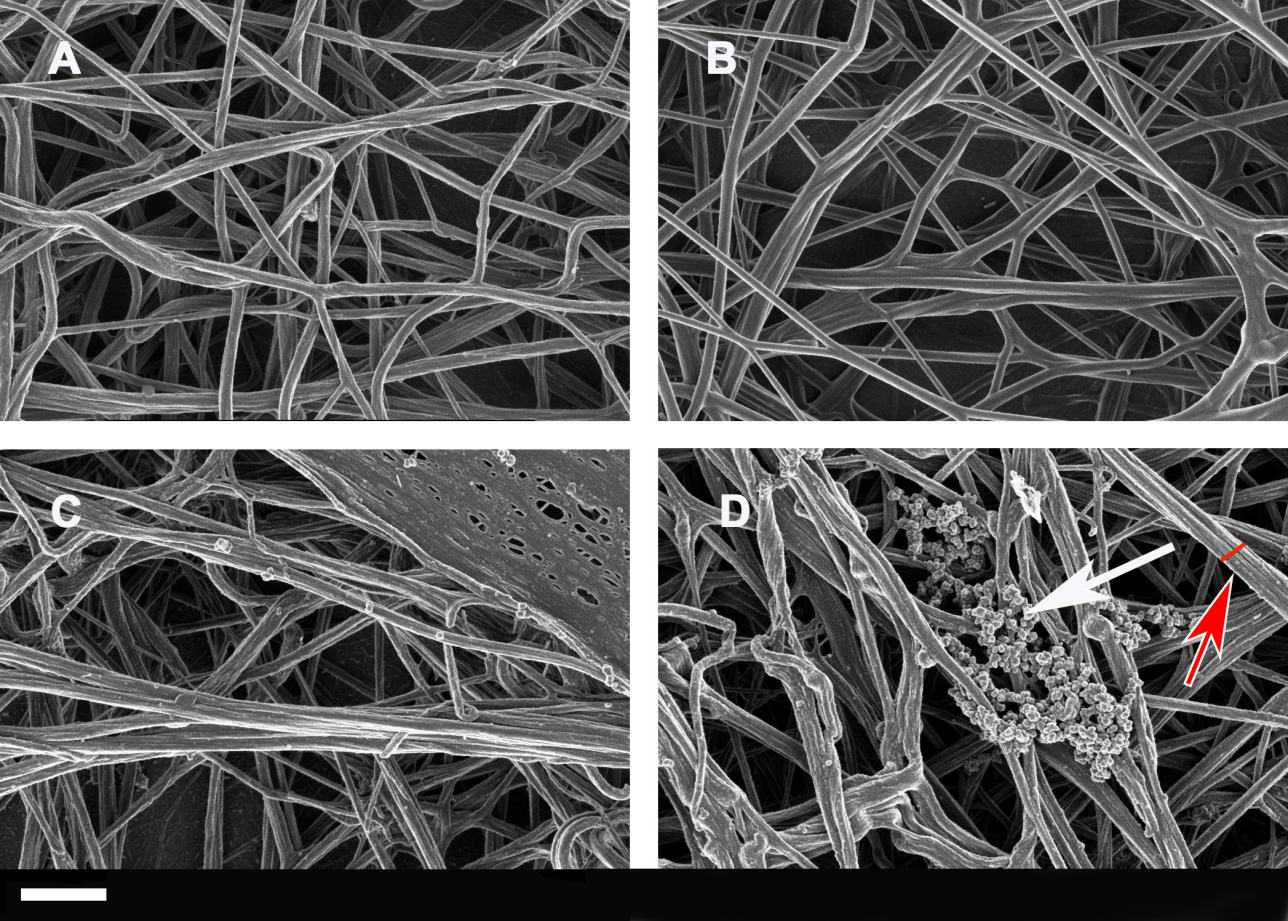
Extensive fibrin networks from two healthy individuals (A and B) and from two Alzheimer-type dementia individuals (C and D) created by adding thrombin to platelet poor plasma White arrow possibly shows LPS or coagulated plasma proteins, red arrow and red line shows thicker fibrin fiber. Scale bar: 1 μm

Due to the observations that the viscoelastic properties of clot generation were significantly changed as judged using the TEG®, SEM was performed with both whole blood and PPP. Figure 3 shows SEM micrographs from whole blood and PPP of healthy individuals, while Figures 4 and 5 shows examples from AD individuals. Whole blood from healthy individuals typically shows discoid erythrocytes (RBCs), with platelets that are typically discoid, without pseudopodia and a few that are slightly activated, usually due to contact activation [53, 160, 163–165] (Figure 3A). This slight activation, with resulting pseudopodia formation, is due to contact activation [166]. This ultrastructure is in line with our database of thousands of micrographs from healthy individuals. Although, for the current study we used age- and gender-matched healthy individuals, there are not great variations between RBCs and fibrin networks of young and old individuals when they are healthy. RBCs are typically discoid and fibrin fibres typically are seen as individual strands.

Figures 4 and 5 show extensive fibrin networks from whole blood and PPP for AD individuals. Recently we noted the presence of bacteria inside and around RBCs of AD patients [90]. Figure 4D also shows an example of an eryptotic erythrocyte. The term eryptosis is a type of suicidal death of erythrocytes, that was discovered recently. It is characterized by erythrocyte shrinkage, blebbing, and phospholipid scrambling of the cell membrane. For a detailed discussion on eryptosis see various publications by Lang and Quadri [167] [168] [169, 170]. Previously it was reported in AD, that Amyloid peptides may induce RBC eryptosis.

In the current, newly collected samples we also found this phenomenon in whole blood of all individuals from our AD sample (Figure 4). Due to the descriptive nature of SEM analysis, any kind of exact estimation of bacterial numbers per volume blood is unfortunately not possible with this method. Platelets were overactivated and showed extreme spreading, as typically seen during systemic inflammation [166, 171, 172].

Due to the presence of (visible) bacteria in whole blood smears, (as seen with electron microscopy) from AD individuals and therefore potentially the presence of cell wall material that they shed, LPS may be one of the culprits, or at least contribute to the hypercoagulability in AD.

**Figure 6:**
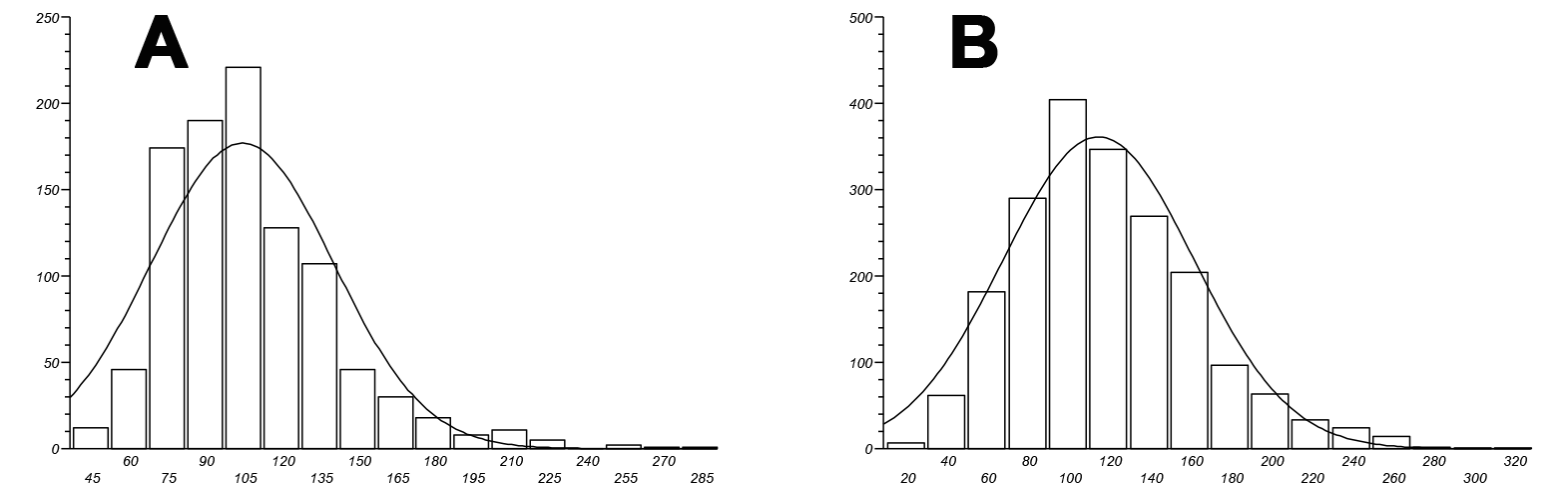
Fibrin fibre thickness (nm) distribution width for both healthy A and Alzheimer-type dementia individuals **B.**

Figure 5 shows fibrin fibre networks from two healthy individuals (A and B) and two AD individuals; the networks are created by adding thrombin to plasma. We also performed measurements on the fibrin fibre diameters using ImageJ (ImageJ is a public domain, Java-based image processing program developed at the National Institutes of Health: http://rsbweb.nih.gov/ij/); Table 2 shows the median fibrin fibre diameter and statistical analysis for the healthy and AD individual fibrin fibres (see Figure 6). Previously we noted a significant difference in fibrin fibre thickness in a pilot study where we measured fibrin fibre thickness in platelet rich plasma (PRP) from AD patients with normal and high serum ferritin values [88]. With regard to median fibrin fibre width, the value for the healthy (younger) subjects was 105± 3 nm [58]. These measurements were taken on samples from younger individuals. The high ferritin AD group had a fibre width that was significantly higher than the normal ferritin AD group, with 34% less than the high ferritin group [88]. The current results showed that the median fiber thickness of older healthy individuals was 110 nm (± 34 nm) and the median AD fibre thickness was 115 nm (± 44 nm). There seems to be a linkage between fibre diameter and serum ferritin levels, which were not different in the present samples.

### GTT^®^ and TEG^®^ analysis with added LPS

Due to the presence of bacteria in the whole blood of AD individuals as seen in the SEM images, we simulated the potential presence of LPS in naïve whole blood from healthy individuals by adding it. A very low final concentration of 0.2 ng.L^−1^ caused a hypercoagulable state using the TEG^®^ in naïve whole blood when the LPS was added. The TEG^®^ measures the activity of the whole coagulation pathway. The following two parameters were significantly changed to a more hypercoagulable state in when LPS was added to the blood of the healthy individuals:

R value (reaction time in seconds) is shorter with added LPS, showing the time of latency from start of test to initial fibrin formation is decreased.

Time to maximum rate of thrombus generation (TMRTG) is also shorter with added LPS, showing a lowering of the time interval in seconds before maximum velocity of clot growth.

This suggests that added LPS causes quicker and firmer clot generation.

Results from the GTT^®^ showed that added LPS did not have an effect on platelet shear force in the GTT^®^ (seen Table 2). As mentioned previously, this GTT^®^ test measures platelet reactivity (occlusion time, OT), where an OT of less than 300 seconds indicated platelet hyper-reactivity, while one between 300 and 500 indicates normal haemostatic/platelet activity [140]. The median of the OT of the healthy individuals with and without added LPS were in the normal ranges, suggesting that LPS do not affect platelet activity. The GTT also shows the time to lyse (LT), and normal values are less than 2000 seconds. Table 2 shows the median for the healthy individuals are less than 2000 seconds, and that LPS does not cause low thrombotic activity.

One interpretation is that LPS possibly binds (due to its very lipophilic nature) to plasma proteins involved in the coagulation cascade, resulting in the hypercoagulability of naïve whole blood with added LPS. This is supported by the TEG^®^ results. However, measuring OT and LT with the GTT, we see no significant changes between naïve whole blood with and without added LPS (see Table 2). This suggests that LPS does not affect platelet activity.

## DISCUSSION

In the current sample we see that the following parameters were significantly different between healthy and Alzheimer-type dementia individuals:

- Iron (free, is less in AD)
- Transferrin (is less in AD)
- Maximum rate of thrombus generation (MRTG),
- Time to maximum rate of thrombus generation (TMRTG) which is the time interval (s) observed before maximum velocity of clot growth,
- Time taken to achieve final clot strength i.e. amplification (K)
- Alpha or Angle representing the speed at which the fibrin build-up and crosslinking of the fibrinogen fibres takes place.
- Fibrin fibre thickness

These results are indicative of increased coagulability in AD, suggesting structural changes in the physical fibrin fibre packaging. The presence of bacteria in whole blood in AD individuals, as seen with SEM as shown in this paper, suggest that LPS, known to be shed from (Gram-negative) bacterial membranes may play a role in the increased coagulability that is seen in AD PPP.

We assessed coagulation of naive blood with and without LPS. TEG^®^ of naïve healthy blood with added LPS showed that it was indeed the case that LPS affects coagulability, as both the

- R value and
- Time to maximum rate of thrombus generation (TMRTG)

showed increased coagulability (due to a shorter time to maximum rate of thrombus generation) and also shorter initial clot formation, where R is also shorter with added LPS. This suggests that added LPS causes quicker and firmer clot generation. This is in line with the hypercoagulability seen in AD individuals. Due to the very variable and often unreliable currently available measurements for LPS in whole blood (for a review see [17], basal LPS levels were not measured in the samples. GTT, which measures only platelet activity, was not affected by the added LPS. These results suggest that LPS does not directly impact platelet function (at least in a controlled experimental environment).

LPS is known to induce inflammation via cytokine activation [173–175], and a characteristic of inflammation is almost always a hypercoagulatory state [58, 176–183]. LPS may also cause hypercoagulation via tissue factor-(TF-) mediated activation of hemostasis in whole blood samples from adults and neonates [184]. TF is also a cytokine. We suggest that there is a third possible route of acute activation by directly binding to plasma proteins involved in the coagulation cascade to cause hypercoagulation. The results presented here support such an acute (fast) reaction.

These results suggest that the clot in AD forms faster, resulting in a firmer clot, reflected in both a decreased time to clot formation and time to maximum firmness of clot. The reason for this changed fibrin structure may be due to a changed fibrinogen packaging during the clot formation, and this changed packaging may be due to the presence of LPS. Future work will seek to establish this directly.

## MATERIALS AND METHODS

### Volunteer details and blood collection

Blood samples were obtained from non-smoking Alzheimer-type dementia (AD) patients, identified by a Neurologist and under the care of a medical practitioner. Specifically, care was taken to exclude vascular dementia. We also recruited “healthy” age-matched individuals that did not smoke. It should be noted that the term “healthy” is used in this paper to describe an individual that has does not have dementia. Ethical clearance was obtained from the Health Sciences Ethical committee from the University of Pretoria, and informed consent was obtained from family members who act as carers of the patients. Healthy individuals also filled in consent forms. Blood was collected in two x 4mL citrate tubes, one EDTA tube, and one 4mL clotting tube for iron level determination. This collection and all handling of samples were performed under very strictly aseptic conditions, in order to prevent any microbial contamination of samples.

### Statistical analysis

The non-parametric Mann-Whitney U test was adopted to determine P-values using the software StatsDirect (www.statsdirect.com). A p-value of less than 0.05 was considered statistically significant (but cf. e.g. [147, 148]).

### Iron tests

Serum ferritin was measured by using the Bio-Rad Laboratories’ QuantImune ferritin IRMA kit that is a single-incubation two-site immunoradiometric assay. In this IRMA, which measures the most basic isoferritin, the highly purified I-labeled antibody to ferritin is the tracer and the ferritin antibodies are immobilized on polyacrylamide beads as the solid phase. Transferrin was measured with the RayBio^®^ Human Transferrin ELISA from RayBiotech. Serum iron was measured with the Iron Assay kit (Colorimetric assay).

### Viscoelastic tests using platelet poor plasma (PPP)

Coagulation parameters, using PPP of patients and healthy individuals, were done using thromboelastography^®^ (TEG^®^). PPP was brought to room temperature and 340 μl was placed in a disposable cup in a computer-controlled TEG^®^ hemostasis system (Model 5000, Hemoscope, Niles, IL), with addition of 20 μl CaCl_2_ as the last step to initiate clotting. Thrombelastographic data were collected until maximum elastic modulus (MG) is reached or 60 min had elapsed [79, 88, 149–152]. See Table 1 for the parameters that can be obtained when both plasma and whole blood are studied using the TEG^®^. TEG^®^ is typically used to determine clot formation and clot strength [85].

### Scanning electron microscopy (SEM) of whole blood and platelet poor plasma (PPP)

At least 30 minutes after the blood was collected, 10 μl of whole blood were placed directly on a glass cover slip, fixed, dehydrated, dried, mounted and coated with carbon according to previously described methods [155]. Platelet poor plasma (PPP) were obtained and frozen at −80°C. After all samples were collected, PPP were thawed and 10 μl mixed with 5 μl thrombin to create an extensive fibrin network. A Zeiss ULTRA Plus FEG-SEM with InLens capabilities was used to study the surface morphology of erythrocytes, and micrographs were taken at 1kV.

### The Global thrombotic test (GTT^®^) and thromboelastography^®^ (TEG^®^) on naïve, uncitrated whole blood with and without added LPS (final LPS concentration 0.2 ng.L-1)

As mentioned above, this GTT^®^ test measures platelet reactivity (occlusion time, OT), where an OT of less than 300 seconds indicated platelet hyper-reactivity, while one of between 300 and 500 indicates normal haemostatic/platelet activity [140]. It also shows the time to lyse or lysis time (LT), where an LT of less than 2000 seconds shows a normal spontaneous thrombolytic activity and LT of 2000 to 4000 seconds shows a reduced thrombolytic activity [156]. Due to the physical constraints of this test (naïve, uncitrated blood is drawn and immediately dispensed into the machine, and also mixed with LPS (final concentration: 0.2 ng.L^−1^ LPS), and incubated for 3 minutes, only the blood of 10 healthy individuals who came into the laboratory for the tests, were used. LPS from *Escherichia coli* O111:B4 was purchased from Sigma, product number L 2630. Naïve and LPS-treated naïve healthy blood was also tested with the TEG^®^, without added CaCl_2_. These tests were done to determine if LPS causes either (1) platelet-induced hypercoagulability or (2) fibrinogen-induced hypercoagulability in healthy blood. This was done to simulate LPS effects, as our hypothesis states that the hypercoagulability seen in AD, together with the presence of increased serum ferritin and low transferrin levels [157], is related to the presence of bacteria in sterile blood, and that these bacteria are visible using SEM.
